# New frontiers in applied veterinary point‐of‐capture diagnostics: Toward early detection and control of zoonotic influenza

**DOI:** 10.1111/irv.12648

**Published:** 2019-07-23

**Authors:** Daniel Schar, Pawin Padungtod, Nguyen Tung, Michael O’Leary, Wantanee Kalpravidh, Filip Claes

**Affiliations:** ^1^ U.S. Agency for International Development Bangkok Thailand; ^2^ Spatial Epidemiology Lab (SpELL) Université Libre de Bruxelles Brussels Belgium; ^3^ Country Office for Viet Nam Food and Agriculture Organization of the United Nations Hanoi Viet Nam; ^4^ Department of Animal Health Ministry of Agriculture and Rural Development Hanoi Viet Nam; ^5^ U.S. Agency for International Development Hanoi Viet Nam; ^6^ Regional Office for Asia and the Pacific Food and Agriculture Organization of the United Nations Bangkok Thailand

**Keywords:** epidemics, influenza, point‐of‐care testing, public health surveillance

## Abstract

Among the chief limitations in achieving early detection and control of animal‐origin influenza of pandemic potential in high‐risk livestock populations is the existing lag time between sample collection and diagnostic result. Advances in molecular diagnostics are permitting deployment of affordable, rapid, highly sensitive, and specific point‐of‐capture assays, providing opportunities for targeted surveillance driving containment strategies with potentially compelling returns on investment. Interrupting disease transmission at source holds promise of disrupting cycles of animal‐origin influenza incursion to endemicity and limiting impact on animal production, food security, and public health. Adoption of new point‐of‐capture diagnostics should be undertaken in the context of promoting robust veterinary services systems and parallel support for operationalizing pre‐authorized plans and communication strategies that will ensure that the full potential of these new platforms is realized.

## 1 CURRENT LANDSCAPE AND CHALLENGES

Global population growth and shifting dietary preferences in transitioning economies are driving surging demand for animal‐source nutrition. To meet this demand, swine and poultry production in low‐ and middle‐income countries have experienced substantial growth rates.^1^ Absent planned livestock sector development structures, animal production, distribution, and marketing capacities have evolved under variable conditions, presenting substantial emerging zoonotic disease vulnerabilities. Foremost among them is the threat of animal‐origin influenza of pandemic potential.

Following the re‐emergence of highly pathogenic avian influenza A/H5N1 in 2003, Asia witnessed repeated incursions of novel influenza subtypes, notably influenza A/H7N9 that emerged in China in 2013.^2^ Simultaneously, antigenic drift of endemic viruses such as H5N1 and recombination events—including an influenza A/H5N6 carrying the hemagglutinin gene from H5 clade 2.3.4.4—have given rise to new threats.^3^, ^4^, ^5^ Other endemic influenza A viruses—H9N2, for example^5^—continue to circulate, often asymptomatically or subclinically in poultry, adding depth to the pool of genes that may give rise to a future pandemic‐capable influenza strain.

Targeted, risk‐based active surveillance aligned to animal production context, and seasonal and value chain risk—and complemented by passive, event‐based surveillance—has substantially advanced capacities for early detection of animal‐origin influenza.^6^, ^7^ Despite these advances, however, significant lag time from sample collection to transport and analysis at national veterinary diagnostic laboratories has placed the goal of immediate detection and containment of animal‐origin influenza out of reach. Indeed, lack of a rapid, real‐time, accurate, and affordable diagnostic that can be deployed in the field for immediate action remains a chief limitation to zoonotic influenza prevention and control.

Given these limitations, response postures remain perpetually reactive, enabling continual cycles of incursion to endemicity that elevate pandemic emergence risk and present substantial economic, agricultural productivity, and food security impacts in the most vulnerable populations.

## 2 THE POTENTIAL OF RAPID, POINT‐OF‐CAPTURE (POC) DIAGNOSTICS

New advances in molecular diagnostics have paved the way for affordable, highly sensitive and specific point‐of‐capture diagnostics.^8^ The downscaling in size of nucleic acid extraction and high‐speed real‐time PCR platforms is opening new opportunities for utility at or near the source of collection.^8^, ^9^ In many cases, advancements in developing rapid, point‐of‐capture real‐time PCR platforms have been pioneered for veterinary applications, facilitating diagnosis and treatment in companion animal medicine as well as management and control of infectious disease affecting livestock production.^8^, ^10^, ^11^

In the current issue, Inui and colleagues describe the analytical and diagnostic sensitivity and specificity of a portable nucleic acid extraction and real‐time insulated isothermal RT‐PCR platform, and its utility in accelerating upstream influenza detection in poultry in the context of influenza A/H7N9 surveillance in Viet Nam.

The POCKIT Micro iiRT‐PCR and taco extraction platform (GeneReach) consists of two, portable, battery‐operated instruments, enabling testing of samples at or near the site of collection. Reagents do not require refrigeration and can be held at ambient temperatures, eliminating the need for cold chain maintenance. Positive or negative results are available within two hours. In contrast, traditional surveillance approaches that rely on transport of samples to the national or regional reference laboratories in Viet Nam require a minimum of 60 hours, thus shortening by an order of magnitude the diagnostic lag time between collection and result.

In Viet Nam, the Ministry of Agriculture and Rural Development, Department of Animal Health (DAH), piloted the POCKIT Micro iiRT‐PCR platform (GeneReach) in four provinces for early detection of influenza A/H7N9 during the high‐risk season between November 2017 and April 2018. With support from the Food and Agriculture Organization of the United Nations and the US Agency for International Development, DAH and provincial staff collected oropharyngeal swabs from poultry at eight vendor stalls selected at random—pooling five birds/vendor—in each of 17 live bird markets twice weekly across the four provinces. Analysis involved two, four‐well POCKIT Micro iiPCR (GeneReach) machines at each site for simultaneous analysis of all samples collected at the site.

In the context of the influenza A/H7N9 surveillance in Viet Nam, optimal cost efficiency was achieved through RNA extraction, at four USD, on each pool of five birds. Extracted RNA can be used for more than one RT‐PCR, and each reaction was 10 USD. The minimum cost totaled 14 USD for each pool of five birds screened for H7, with additional 10 USD per additional target—for example, N9—tested. In principle, and notwithstanding sample collection and hardware expenses, the H7N9 surveillance approach utilized was budgeted at between 112 and 192 USD per market at each sampling event. New plug‐and‐play platforms combining RNA extraction with RT‐PCR in a single machine are now commercially available. For example, the POCKIT Central platform (GeneReach), with running cost of 12 USD per sample, further reduces the time from single‐sample insertion to result to less than 90 minutes.

Achieving such order‐of‐magnitude reduction in time from sample collection to result presents a series of compelling opportunities in reducing the threat from emergent animal‐origin influenza of pandemic potential. First, diagnostic turnaround measured in hours permits sampling of highest risk populations—in live bird markets or high‐risk farms with suboptimal biosecurity or wild bird contact, for example—and immediate implementation of control and containment strategies, before animals have moved from the premises, thereby limiting onward transmission risk. A positive detection under such conditions permits authorities to operationalize a pre‐planned set of emergency control measures—from movement restrictions and epidemiological analyses of flows into and out of the site to deployment of communication and risk avoidance messaging, and, as warranted, humane depopulation, compensation, site closure, disinfection, and ring vaccination.

Second, early detection limits the magnitude of infection in at‐risk livestock populations, presenting an attractive economic return on investment in avoiding loss associated with widespread transmission and emergency response.^12^

Third, rapid identification of zoonotic influenza permits targeted, animal value chain‐linked human syndromic surveillance at the human‐animal interface. A coordinated response with human health authorities provides an opportunity to rapidly identify influenza‐like illness among in‐contact individuals both at and along value chains and populations linked to the site of detection.

Finally, early detection of novel animal‐origin influenza holds promise of interrupting the repeated cycle of incursion to endemicity. Although the exchange of influenza viruses across the wildlife‐livestock‐human interface may persist, targeted, risk‐based surveillance complemented by rapid PCR diagnostics and a holistic approach to control offer unprecedented options for mitigating impact.

## 3 REALIZING THE POTENTIAL OF EARLY DETECTION, EARLY CONTROL

The application of PoC diagnostics, while promising, must be seen as a component of—rather than a substitute for—foundational veterinary sector services strengthening.

Translating rapid on‐site detection into actionable containment requires sufficient resources, capacities, and commitment to acting on disease intelligence. Advance planning, protocols for data distribution, clear stakeholder roles and authorities, training of on‐site staff, development of communications materials, and periodic capacity testing through simulation exercises are essential. Incorporating such elements of these new PoC diagnostics into existing national zoonotic influenza control strategies and emergency operations center plans may be an effective means of leveraging multiministerial relationships from national to local levels.

Current limitations include a requirement for pathogen surveillance to an identified target. Fieldwork using the rapid, iiRT‐PCR platform described was explicitly developed to quickly identify a possible cross‐border incursion of A/H7N9 into Viet Nam. A lengthier, two‐step assay involving conserved influenza A target–matrix protein, for example—followed by priority or OIE‐reportable subtypes (H5, H7, and H9) is also feasible. On‐site detection of a novel influenza subtype outside of this framework remains a challenge until a rapid, portable pan‐influenza platform is developed mirroring the one‐step pan‐influenza A virus benchtop PCR primer and probe sets that have been described.^13^

Other limitations encountered include a need to develop surge capacity policies enabling rapid resource reallocation toward containment, and data management systems facilitating an accelerated diagnostic‐to‐response time frame. Confidence in the validity of the diagnostic result is a further pre‐requisite for adoption, and early engagement of involved stakeholders—particularly those with financial and livelihood interests at stake—is critical.

## 4 SCALING TO OTHER DISEASE SURVEILLANCE AND CONTROL APPLICATIONS: NEW FRONTIERS IN DISEASE CONTROL

Additional studies will need to validate these platforms in differing geographic contexts and with influenza subtypes. Conceivably, they hold utility for rapidly identifying and controlling other priority transboundary animal disease pathogens, including viral etiologies of porcine reproductive and respiratory syndrome (PRRS), classical swine fever (CSF), African swine fever (ASF), and emerging coronaviruses with a livestock transmission interface.^14^ The August 2018 notification by China to international authorities concerning the detection of African swine fever virus in domestic swine^15^ across multiple provinces presents an opportunity to trial such PoC diagnostic platforms at scale in a region that represents a substantial portion of the global swine production industry. Additional targets could include emerging viral zoonoses—such as the Middle East respiratory syndrome (MERS) coronavirus—where animals may be mildly or asymptomatic and clinical diagnoses are not readily distinguishable.^16^

Similarly, studies assessing the economics of employing PoC diagnostics at scale are needed, particularly in the context of alternative investments, including in preventative interventions, such as enhanced farm biosecurity, vaccination coverage, and improved management of disease control along highest risk value chains.

As the incidence of bacterial multidrug resistance accelerates, PoC diagnostics could be a valuable addition to addressing the threat from antimicrobial resistance (AMR). Future applications could see species‐specific, multiplex panels that inform improved production and management, thereby reducing empirical use of broad‐spectrum antimicrobials in animal production and ​scaling​ back pressures driving AMR.^17^

Finally, application of these PoC diagnostics in control of endemic disease should also be further explored, as they may prove useful in structuring compartmentalization toward “freedom from disease” status.

The holistic use of nascent PoC technologies holds promise of disrupting the cycle of zoonotic influenza incursion to endemicity, and ushers in a new era of disease detection and prevention with broad benefits to animal and human health, livelihoods, food security, and nutrition.
